# FloBuzz: A modular feeder system for automated aversive stimulation in free-flying bees

**DOI:** 10.1371/journal.pone.0361207

**Published:** 2026-10-26

**Authors:** Elvin Gultekinoglu, Babur Erdem, Atakan Botasun, Okan Can Arslan, Sedat Sevin, Ayse Gul Gozen, Hande Alemdar, Erol Sahin, Ali Emre Turgut

**Affiliations:** 1 Department of Mechanical Engineering, Middle East Technical University, Ankara, Türkiye; 2 Center for Robotics and Artificial Intelligence (ROMER), Middle East Technical University, Ankara, Türkiye; 3 Department of Pharmacology and Toxicology, Faculty of Veterinary Medicine, Ankara University, Ankara, Türkiye; 4 Department of Biological Sciences, Middle East Technical University, Ankara, Türkiye; 5 Department of Computer Engineering, Middle East Technical University, Ankara, Türkiye; University of Alberta, CANADA

## Abstract

Understanding how environmental stressors affect pollinator behavior is essential for assessing ecosystem health. Automated flower and robotic feeder systems (AFRFSs) have transformed studies of pollinator foraging and learning, yet most remain reward-centric and cannot probe aversive learning under field conditions. Here, we present FloBuzz, a modular AFRFS that combines automated reward delivery with computer vision visit detection to trigger closed-loop electric shock stimulation in free-flying honey bees (*Apis mellifera*). The system consists of a 3D-printed feeder with a shock grid, a syringe pump with fluid level feedback, and a computer vision based shock module. In a replicated field proof-of-concept experiment, mean bee occupancy decreased with increasing stimulus voltage, demonstrating a measurable voltage-dependent deterrent response. These results validate the ability of FloBuzz to deliver automated, programmable aversive stimulation under outdoor conditions. Its modular architecture provides a flexible platform for future studies requiring controlled integration of appetitive and aversive stimuli in freely foraging bees.

## 1. Introduction

Pollination is one of the most critical factors for plant survival, and bees, in particular, are considered keystone species for ecosystem sustainability and agricultural productivity [1]. Therefore, studying bee behavior is necessary for both environmental sustainability and agriculture. Automated flowers and robotic feeder systems (AFRFSs) are essential tools for improving experimental conditions for pollinators [2]. Here, we introduce FloBuzz, an AFRFS that delivers automated aversive stimulation to free-flying bees under outdoor conditions. Aversive conditioning examines an organism’s ability to learn associations involving a negative stimulus [3] and represents a potential application of this platform.

AFRFSs are essential experimental tools that enable researchers to study the mechanisms underlying pollinator behavior, learning, and floral trait evolution under controlled, replicable conditions [2,4]. Compared to natural flowers, AFRFSs offer superior control over reward timings and floral cues such as color, shape, scent, and nectar concentration, thereby mitigating challenges in foraging experiment design. This level of control makes AFRFSs invaluable for dissecting how pollinators integrate multimodal cues during decision-making and for quantifying foraging efficiency across sensory modalities [5–7]. Additionally, working with real flowers poses substantial logistical challenges due to correlations between floral traits [8], high maintenance costs [2], and numerous uncontrolled environmental variables [9]. Natural variations in nectar chemistry, volatile release, or petal morphology can bias behavioral interpretations [10]. Therefore, AFRFSs provide consistency across experimental replicates and eliminate this bias. Moreover, AFRFSs can repeatedly present controlled combinations of visual, olfactory, and reward cues in response to animal visits [4,11]. In previous studies, the utility of AFRFSs has been expanded for behavioral ecology and neuroethology by enabling long-term experiments [12], real-time monitoring [13], and standardized reward delivery [14].

Furthermore, foraging decisions in natural environments are shaped not only by rewards but also by risks, highlighting the need for AFRFSs that can integrate aversive or nociceptive stimuli alongside controlled rewards. Integrating nociceptive stimuli and predator dummies into AFRFSs enables the investigation of aversive learning. For example, imitating sit-and-wait predators such as mantids or crab spiders as aversive elements suppresses honey bee foraging [15–17]. Other methods apply nociceptive stimuli, such as heating [18,19].

Electric shock is another nociceptive stimulus in the electric shock avoidance assay, which is a standard laboratory procedure. It has been utilized to investigate the role of chemicals [20,21] and neuromodulators [22], caste specificity [23], odors and colors [24], and neural pathways [25] on learning. Furthermore, the electric-shock avoidance assay has recently been adapted for free-flying bumblebees in indoor settings [26].

We have devised FloBuzz to adapt the standard laboratory electric shock avoidance assay for use in outdoor environments. Through its computer-vision-based visit detection and trigger module, the system delivers shocks in user-defined continuous, intermittent, or delayed patterns. FloBuzz also provides food solutions as appetitive stimuli alongside these aversive triggers. Thus, FloBuzz integrates various engineering approaches to combine the well-established method of electric shock avoidance with appetitive stimuli through an automated system, enabling researchers to conduct studies in natural environments. Consequently, FloBuzz is bound to facilitate research on learning, foraging, predatory incidence, and risk assessment in a natural environment for free-flying honey bees.

## 2. Materials and methods

### 2.1. System architecture of FloBuzz

We designed FloBuzz to support controlled behavioral experiments by integrating stimulus (electric shock) delivery via computer-vision-based triggering, and automated food supply as a reward into a single modular system (Fig 1). The system consists of a feeder module, the In-Trigger module, and a syringe pump with a control unit (Fig 2). The feeder module is the primary component that enables interaction with bees. An electric shock grid in the feeder module is connected to a shock switch, which is controlled by the In-Trigger module. The feeder module is attached to the syringe pump via a flexible food delivery tube.

**Fig 1:**
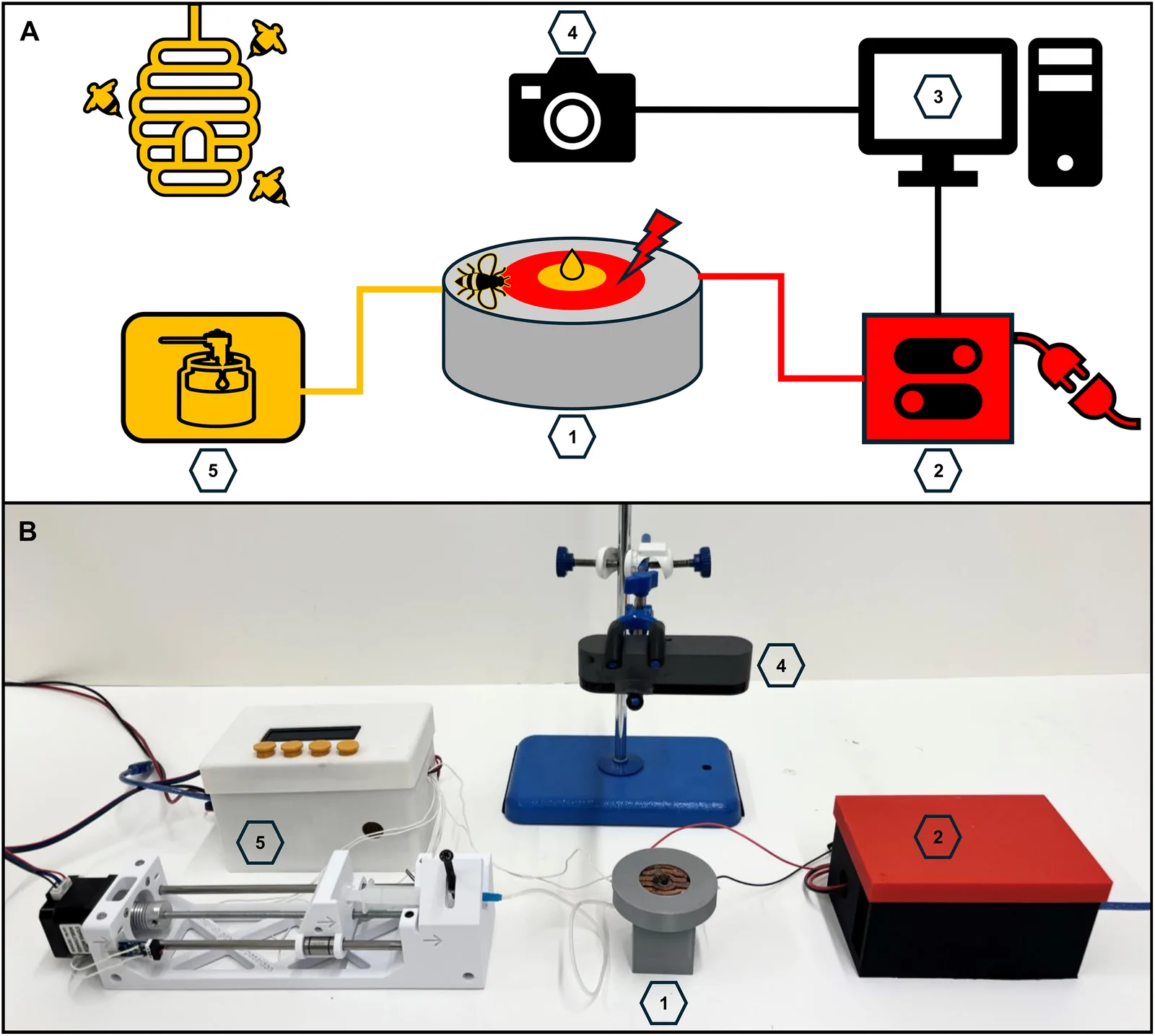
View of the experimental setup. (A) The schematic illustration of the experimental setup. (B) The picture of the experimental setup. (1) The feeder module provides the food solution to the bees and acts as the platform where the electric shock is applied. (2) The shock switch triggers an electric shock based on control signals from the In-Trigger module. (3) The computer runs the In-Trigger module and processes visual input from the webcam. (4) The webcam acquires visual information from the feeder module for visit detection. (5) The syringe pump supplies the food solution to the feeder module, regulated by a control unit.

**Fig 2:**
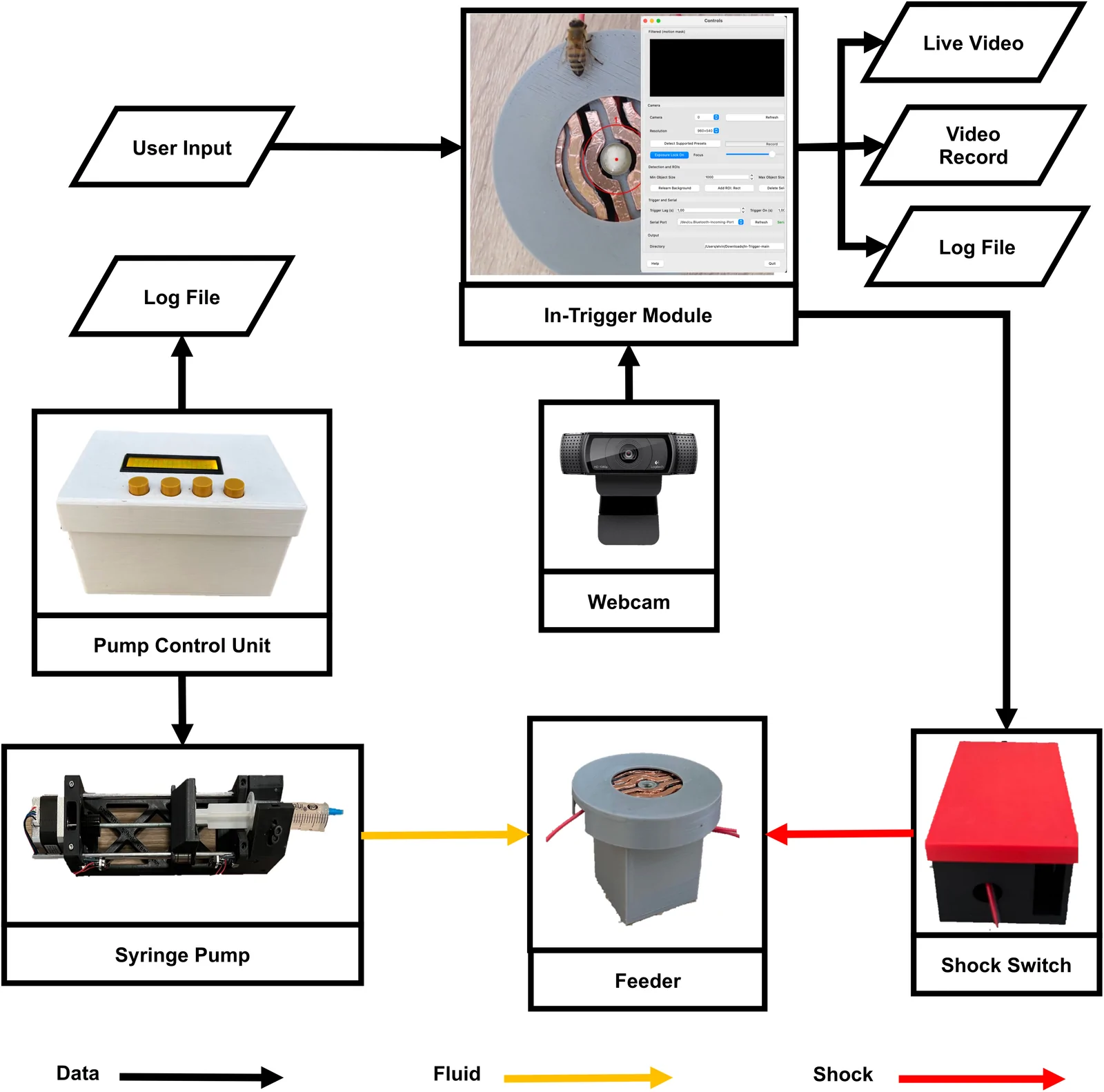
Low-level representation of modules and their interactions in the FloBuzz system. The feeder module is connected to the syringe pump and the shock switch. The shock switch is controlled by the In-Trigger module and enables the activation and deactivation of the electric stimulus. Visual information from the feeder module is acquired by a webcam and transmitted to the In-Trigger module. The pump control unit governs the operation of the syringe pump, which supplies the food solution to the feeder module continuously.

The In-Trigger module detects objects entering predefined regions of interest (ROI) within the camera frame using computer vision algorithms. It activates the shock switch with user-defined delays and durations.

The syringe pump delivers a steady supply of food solution to the feeder module. Its design was adapted from the open-source Poseidon syringe pump [27]. A pump control unit (described in the S1 Appendix) enables the pump to automatically refill the feeder as bees consume the solution. A script running on this subsystem monitors solution levels and logs each refilling event.

### 2.2. Feeder module

The feeder module serves as the physical interaction interface between the bees and the FloBuzz system, providing both the food reward and the aversive electric stimulus. The feeder module is composed of four parts that fit together: a main base, a cap, an electric shock grid, and a collecting box (Fig 3).

**Fig 3:**
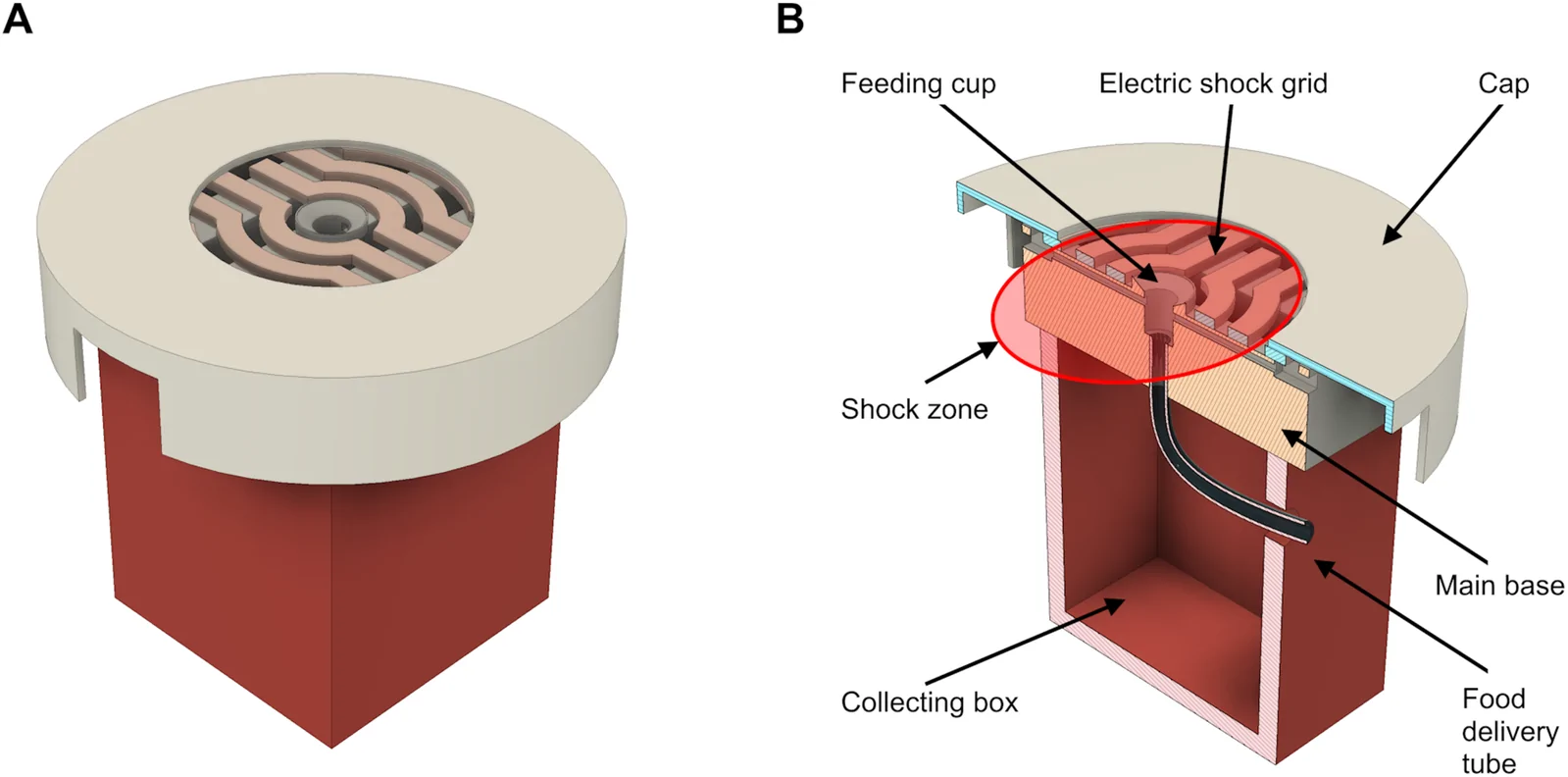
Schematic of the feeder module. (A) Technical drawing of the feeder module. (B) The sectional view. The main base includes the feeding cup. The food delivery tube is penetrated to the main base and it enables controlled food solution delivery. The electric shock grid surrounds the feeding cup and displays the shock zone. The cap is positioned above the grid and it defines the landing area. The collecting box beneath the assembly provides a large internal cavity to capture any overflow of the food solution.

In the module, the main base houses the feeding cup and includes holes for silver-plated feedback cables. These cables function as electrodes and provide analog readings to an Arduino board mounted on the custom printed circuit board (PCB) (S1 Fig). The feedback cable lines are also interfaced with the pump control unit to enable closed-loop actuation of the syringe pump during automatic refilling. The feedback algorithm monitors the food solution and triggers automatic refilling when the cup is empty by evaluating analog readings against an experimentally determined threshold value. The feeding cup is positioned at the center of the main base and functions as the reservoir for the food solution. The feeding cup geometry features a gentle slope and edge protrusions to minimize surface tension and prevent overflow. The feeding cup is 4.5 mm in diameter and 5.3 mm deep; these dimensions prevent bees from falling in while limiting evaporation. Feedback cables are placed 1.2 mm below the rim of the feeding cup, allowing bees to comfortably consume the solution while maintaining continuous monitoring of the solution level. An opening at the bottom of the feeding cup secures a butterfly-type infusion tube, hereafter referred to as the food delivery tube, to enable controlled delivery. The food delivery tube transports the food solution from the syringe pump to the feeding cup of the feeder module.

The electric shock grid delivers the aversive stimulus with a 2.5 mm gap between grid lines. The bees receive a shock when their feet complete the circuit by bridging two separate lines. The electric shock grid can be manufactured by applying copper tape to a 3D-printed grid or by directly cutting a copper plate. The cables are soldered directly to the grid and connected to the shock switch (S2 Fig).

The FloBuzz feeder cap encloses the main base. It features a circular design to ensure the shock area is radially symmetric. This geometry was chosen because bees preferentially visit radially symmetric artificial flowers [28,29]. With a diameter of 68.0 mm, the cap provides ample landing space for the bees. The hole in the center of the cap is 33.0 mm in diameter. This hole exposes the electrical grid, and its radius is approximately the length of a bee, ensuring that the bee will touch the grid in this shock zone. A catch basin beneath the main base captures any overflow, preventing food solution from spilling into the experimental area.

### 2.3. Computer vision-based stimuli trigger module (In-Trigger)

To enable precise, automated aversive stimulus delivery, we developed In-Trigger, a system integrating a video-processing pipeline, a graphical interface, and microcontroller-based actuator control (available from https://github.com/baburerdem/In-Trigger). This integrated module offers a flexible, low-latency, and fully configurable system for closed-loop experimental paradigms that require precise synchronization between behavioral detection and stimulus delivery. The system detects objects entering predefined spatial regions within the camera frame. It activates external stimulus devices with user-defined delays and durations (S3 Fig).

We implemented the core functionality in Python using the OpenCV [30] library for real-time video acquisition and analysis. The script can manage background subtraction, object detection, Region of Interest (ROI) mapping, trigger logic for the shock switch, and data logging. Video is captured from USB cameras via the highest-priority available backend. Motion detection is performed using a *MOG2* [31] background subtraction model. Detected foreground masks are morphologically filtered and intersected with user-defined ROIs. The system tracks individual moving objects by matching detected contours across consecutive frames. It evaluates their position relative to each ROI. A trigger event is confirmed only when a detected object is localized within a specific ROI.

Each ROI is mapped to a unique digital output pin on an Arduino controller, which receives commands via serial communication and switches the stimulus device accordingly. When an ROI trigger is confirmed, a shock event is scheduled on the Arduino controller through a sequence of activation and deactivation commands. Timing parameters, including the delay between detection and trigger onset, and the duration of the active trigger, are configurable via the graphical user interface (GUI).

The GUI developed by *PySide6* enables full control of experimental parameters and system status. Users can select cameras and resolutions, define and edit ROIs interactively on the live preview, toggle horizontal flipping, and adjust autofocus or manual focus settings. Controls also include configuration for detection thresholds (minimum and maximum object size), trigger delays, stimulus durations, and the selection of a serial port for Arduino communication. The GUI provides real-time visual feedback of both raw and processed frames, displaying annotated bounding boxes and ROI outlines (Fig 4).

**Fig 4:**
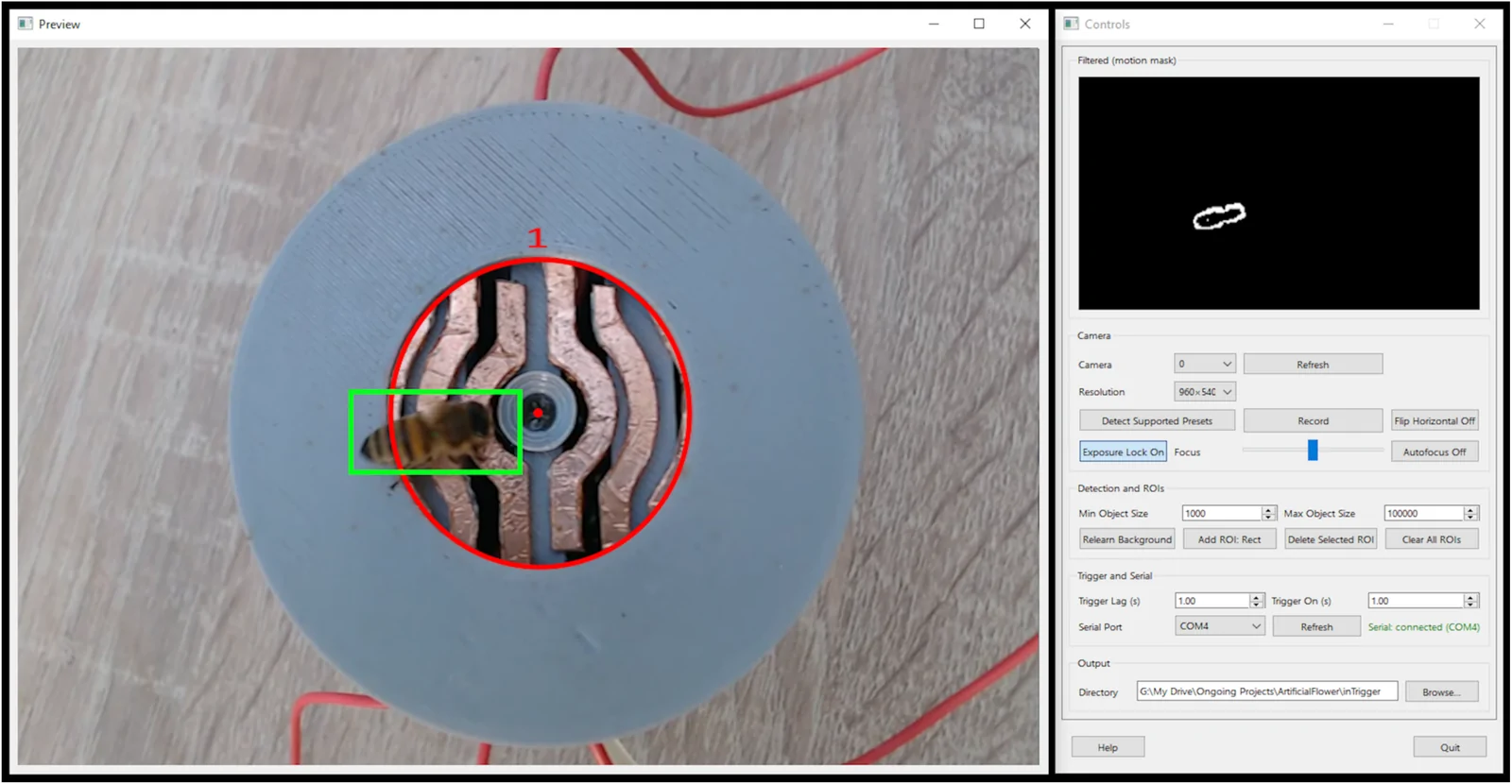
In-Trigger graphical user interface and real-time detection during the experiment. The left panel shows the live camera preview of the feeder module during an experimental session. The circular red region indicates the predefined shock zone. A visiting honey bee is detected and is enclosed by a green bounding box. The right panel displays the In-Trigger control interface, which provides real-time visualization of the filtered motion mask (top) and allows full configuration of experimental parameters, including camera selection and resolution, background relearning, minimum and maximum object size thresholds, trigger delay and on-time durations, serial communication with the shock switch, and data recording options.

All relevant experimental data are automatically logged during recording sessions. Video output is saved in MP4 format, alongside a high-quality JPEG snapshot of the first annotated frame to verify ROI placement. Concurrently, a timestamped plain-text log file is created, detailing frame-by-frame object counts per ROI, shock activation status, frame indices, and the associated video file. These logs ensure traceability and facilitate post-hoc validation of each experimental run.

The electric shock switch is controlled by an Arduino board, which sends on/off commands to the IRF520 driver. An external power source and a MOSFET IRF520 driver module are used as the solid-state switching element. Gate-drive and protective components are implemented, and the switching system is controlled through the In-Trigger GUI (S2 Fig).

Each confirmed detection initiates a stimulation cycle. A detection is confirmed when the centroid of a tracked object remains inside an ROI for three consecutive frames, and this moment is recorded in the log as “Object Detected”. This confirmed detection serves as the temporal reference for the stimulation cycle. The shock grid is then scheduled to activate after the user-defined delay and to remain active for the user-defined stimulus duration. The activation command is sent at the first processed frame following the end of the delay and is recorded as “Shock ON”. Thus, “Object Detected” marks the confirmed detection and “Shock ON” the activation command, after which current reaches the grid with a brief switching latency.

At the end of the stimulus duration, the deactivation command is sent and recorded as “Shock OFF”, which marks the end of the stimulation cycle. While a stimulation cycle is pending or the shock grid is active, further detections in the same ROI are ignored, and the system becomes available to respond to a new visit only after the stimulation cycle ends. A bee that remains continuously within the ROI therefore produces only one confirmed detection; prolonged presence does not generate repeated triggers. A subsequent trigger requires the bee to leave and re-enter the ROI, or for its track to be re-initialized. Once a stimulation cycle has been scheduled, it is not cancelled if the bee leaves the ROI during the programmed delay.

### 2.4. Field experiment

To validate our system, we conducted a field experiment using five colonies at the Middle East Technical University campus. The feeder module, syringe pump, and a camera were located on a table 3 meters away from the hives (Fig 1). The syringe pump was initiated, and the feeder module maintained a 50% (v/v) honey/water solution. Bees were allowed to approach the artificial flower freely. Colonies had access to a single shared feeder and were not tested separately. Bees were unmarked, and neither individual identity nor colony of origin could be determined. The five colonies therefore did not constitute independent colony-level replicates.

Data were collected over five experimental days, from 27 to 31 July 2026. Irradiance, air temperature, and relative humidity were measured using a sensor suite (https://github.com/baburerdem/IrradianceSensor) [32] during each run of the experiment. Across the experimental runs, UV irradiance was 357.66 ± 373.23 µW cm^-2^, temperature was 29.06 ± 2.03 °C, and relative humidity was 35.51 ± 6.35%.

Four voltage treatments were evaluated: 0, 3, 6, and 12 V. The 0 V treatment served as the unshocked control. A midpoint of 6 V was selected based on its use in previous honey bee aversive-conditioning experiments [3,23]. Treatments corresponding to half and twice this midpoint were included to characterize the voltage-dependent response across a broader range.

The In-Trigger module monitored the predefined shock zone and controlled voltage delivery. The system applied direct current at a specified voltage for 4 s, with a 3-s delay following each confirmed detection.

The experiment followed a complete-block design comprising nine blocks and 36 analyzed runs. Each block consisted of one run at each of the four voltage treatments, providing nine run-level replicates per voltage. A block was defined as one complete series of four consecutive voltage-treatment runs conducted under the same experimental session.

Treatment order was varied among blocks using three predefined sequences: ascending (0, 3, 6, and 12 V), descending (12, 6, 3, and 0 V), and non-monotonic (6, 3, 0, and 12 V). Each sequence was used in three blocks. This variation was implemented to mitigate systematic confounding between voltage treatment and temporal changes in bee abundance during an experimental session.

Each run lasted 300 s. Immediately after a run, the artificial flower was covered to prevent bee access. Following an approximately 10-min inter-run interval, the cover was removed, and the next run began. The three ascending blocks were conducted first, followed by the three non-monotonic blocks and then the three descending blocks. Treatment sequences were therefore grouped across the experimental period rather than fully balanced across experimental days. The In-Trigger module recorded each session and controlled shock delivery according to the assigned voltage treatment.

### 2.5. Statistical analysis for voltage-dependent avoidance

Video recordings were scored by two observers, who counted the number of bees occupying the artificial flower every 5 seconds. Thus, each 300-s (5 min) experimental run contained 60 occupancy measurements recorded at 5-s intervals. Starting occupancy was defined as the number of bees present at the first recorded observation of each run (t = 0 s). Mean run-level occupancy was calculated from 60 counts recorded at 5-s intervals from 0 to 295 s. These measurements quantify the number of bees present at the feeder at sampled times. They do not quantify the total number of visits or unique visitors during a run, because individual bees were unmarked and arrivals and departures were not separately enumerated.

A block-adjusted linear dose-response model was fitted to determine whether mean bee occupancy changed systematically with voltage. Voltage was entered as a continuous predictor, and experimental block was included as a fixed categorical effect. This formulation estimated the average within-block change in occupancy associated with a 1 V increase while controlling for differences in baseline bee abundance among blocks. The model was specified as:


$$\begin{array}{c} Mean\text{\textasciitilde{}}bee\text{\textasciitilde{}}occupancy=\beta_{0}+\beta_{1}(Voltage)+Block+\varepsilon \end{array}$$

The voltage coefficient (β_1_) represented the estimated change in mean bee occupancy associated with a 1 V increase after adjustment for block effects.

Regression coefficients were reported with standard errors, 95% confidence intervals, t-statistics, and two-sided p values. Model fit was summarized using R^2^ and adjusted R^2^. Statistical significance was assessed at α = .05.

To assess whether the voltage–occupancy relationship persisted after accounting for treatment order, the block-adjusted model was extended to include run position within each block as a categorical fixed effect with four levels. The response variable remained mean bee occupancy per 300-s run, and voltage was retained as a continuous predictor. The voltage coefficient from this model was compared with that from the original block-only model. Because each block was uniquely identified and occurred within one experimental day, block fixed effects also accounted for differences in average occupancy among blocks conducted on different days or at different positions within a day. Individual block trajectories were plotted in chronological order to show variation among blocks and treatment sequences.

Analyses were conducted in R version 4.4.1 [33]. Data processing, model summarization, marginal prediction, and visualization were performed using the readxl, dplyr, broom, emmeans, and ggplot2 packages.

### 2.6. Benchmark evaluation methodology

The technical performance of FloBuzz was evaluated using the 27 experimental runs conducted at 3, 6, and 12 V. The 0 V control runs were excluded because no electric stimulus was applied.

To evaluate bee detection, the system records were compared with the corresponding video recordings (S1 Movie). Two observers inspected the videos and determined whether a bee was present in the shock zone within ±1 s of each recorded system detection. The ± 1-s window was centered on the “Object Detected” event recorded by In-Trigger. A detection was considered correct when a bee was observed in the shock zone within this window, and false when the system recorded a detection without a corresponding bee presence. Detection precision was calculated as the proportion of system detections that correctly corresponded to a bee presence.

Missed detections were evaluated during the free intervals in which the system was available to respond to a new bee visit. Each confirmed detection initiated a 7-s stimulation cycle, consisting of the 3-s delay followed by the 4-s stimulus. Accordingly, a free interval was defined as the period from the end of a stimulation cycle until the next confirmed detection. A ± 1-s tolerance was applied at both ends of each interval. An interval was classified as missed when an observer identified a bee in the shock zone during the available period and the system did not generate a corresponding detection; otherwise, it was classified as responded when the detection was correct. The miss rate was calculated as the proportion of missed intervals among all intervals in which a bee was confirmed.

The influence of outdoor light conditions on detection performance was evaluated using ultraviolet irradiance recorded during the experimental runs. The relationship between detection precision and average irradiance was tested using Spearman rank correlation.

The timing of electric stimulation was evaluated by measuring the interval between each confirmed detection and the activation command, and the duration of the applied stimulus. These values were compared with the programmed 3-s delay and 4-s stimulus duration. The stability of the applied voltage was assessed using a digital multimeter before and after the experimental runs.

Finally, the reliability of the feeder and overall system was evaluated by confirming successful food delivery, completion of the planned experimental runs, recovery of video and system records, and successful completion of triggered stimulation cycles.

### 2.7. Ethical statement

According to Article 4.b of the Regulation on the Working Procedures and Principles of Animal Experimentation Ethics Committees, published in the Official Gazette of the Republic of Turkey (February 15, 2014, Issue: 28914), an experimental animal is defined as “any non-human vertebrate organism, including free-living or reproducing larval forms, live cephalopods, and mammals from the last third of their normal fetal development, used in procedures.”

Therefore, research conducted with honey bees (*Apis mellifera*) is not subject to evaluation by the Ethics Committee. We also declare that no bee individuals were harmed in our study, and the experiment durations were kept short enough to obtain only the necessary data, minimizing the discomfort experienced by the bees during the experiment.

## 3. Results

### 3.1. Voltage-dependent avoidance

At the first recorded observation (t = 0 s), mean bee occupancy was 1.22 ± 2.22, 0.89 ± 1.45, 0.44 ± 0.73, and 0.22 ± 0.67 bees at 0, 3, 6, and 12 V, respectively (mean ± standard deviation; nine runs per voltage). The subsequent 300-s summaries describe mean occupancy based on repeated counts of bees present, rather than cumulative numbers of visits or unique visitors. Mean bee occupancy declined with increasing voltage. Across the nine experimental blocks, observed mean ± standard deviation occupancy was 6.59 ± 5.10 bees at 0 V, 5.59 ± 4.45 bees at 3 V, 3.94 ± 3.51 bees at 6 V, and 3.61 ± 3.46 bees at 12 V. Thus, the observed mean occupancy at 12 V was 45.2% lower than that recorded at 0 V.

The block-adjusted linear dose–response model identified a significant negative relationship between voltage and mean bee occupancy (β = −0.252 bees V^-1^, 95% CI [−0.453, −0.051], t(26) = −2.57, p = 0.016). Accordingly, each 3 V increase was associated with an estimated reduction of 0.76 bees in mean occupancy. Across the full 0–12 V range, the model predicted a decrease of 3.02 bees. The model, including voltage and block effects, explained 71.2% of the variation in mean occupancy (adjusted R^2^ = 0.613; F(9, 26) = 7.16, p < 0.001).

These findings demonstrate that increasing voltage reduced the mean occupancy of the artificial flower and therefore produced a measurable deterrent response in visiting bees (Fig 5). However, no pairwise comparison reached the adjusted significance threshold of p < 0.05 according to the pairwise voltage comparisons using a categorical-voltage model adjusted for experimental block and categorical run position, with Tukey adjustment for six comparisons.

**Fig 5:**
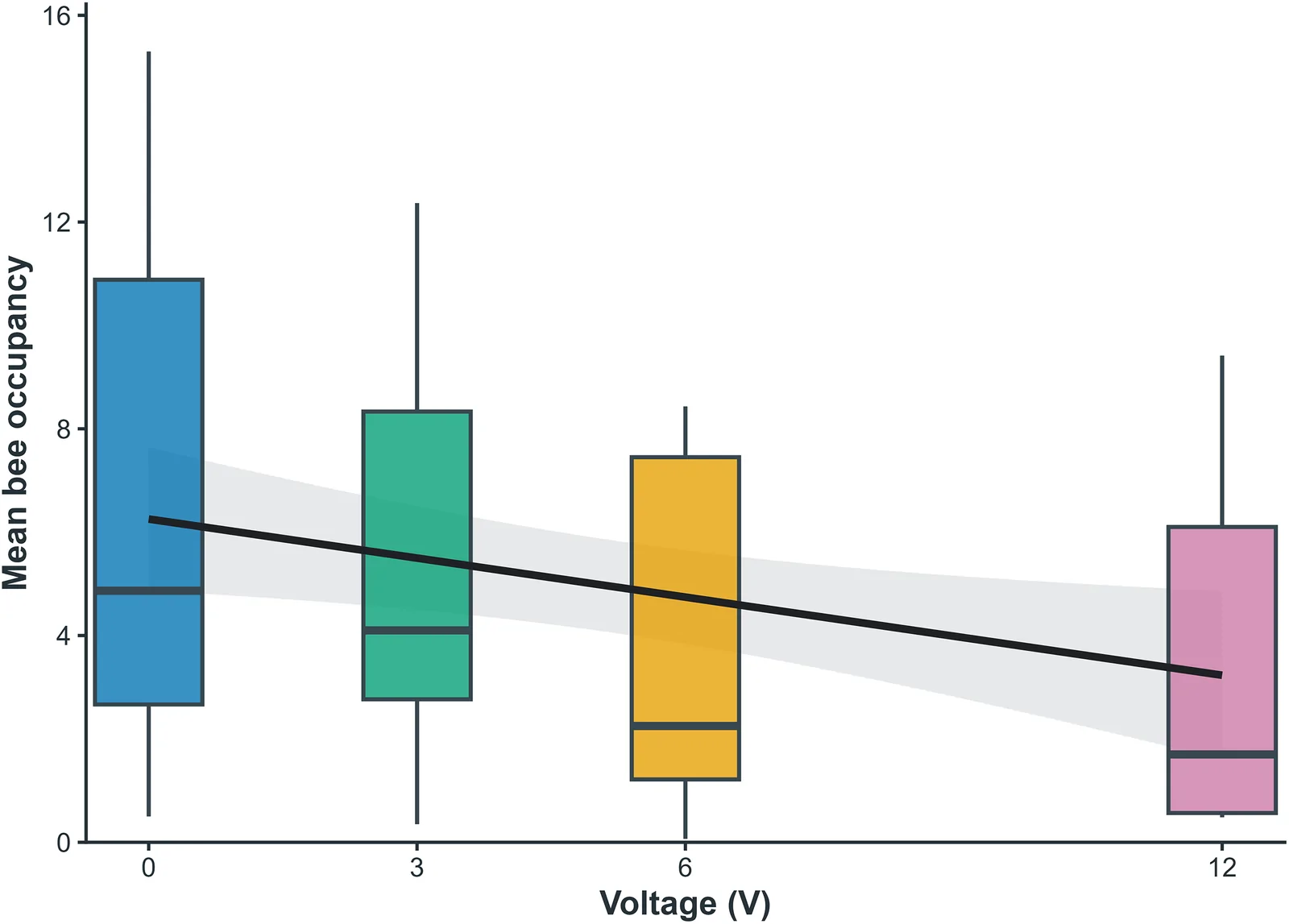
Effect of applied voltage on mean bee occupancy. Boxplots show the distribution of run-level mean occupancy across nine experimental blocks at each voltage. Center lines indicate medians, boxes represent the interquartile range, and whiskers extend to the most extreme values within 1.5 times the interquartile range. Each run-level mean was calculated from 60 occupancy measurements recorded at 5-s intervals during a 300-s experiment. The solid black line represents marginal predictions from the block-adjusted linear dose–response model, with experimental block included as a fixed categorical effect. The grey shaded area indicates the 95% confidence interval of the adjusted trend.

Along with the given results, the negative voltage–occupancy relationship persisted after adjustment for run position within blocks (β = −0.321 bees V^-1^, SE = 0.109, 95% CI [−0.548, −0.095], t(23) = −2.934, p = 0.007). This corresponded to an estimated decrease of 0.96 bees in mean occupancy per 3-V increase and 3.85 bees across the 0–12-V range. Individual block trajectories showed variation in the response: occupancy at 12 V was lower than at 0 V in all ascending and descending blocks, whereas the non-monotonic blocks showed mixed patterns (S4 Fig).

### 3.2. Benchmark evaluation

Across the 27 benchmarked runs, FloBuzz produced 694 detection responses during 135 min of recording. Comparison with the video recordings showed an overall detection precision of 91.9%, with 638 correct detections and 56 false detections. This corresponded to 0.41 false detections per minute.

Among 634 free intervals in which a bee was confirmed to be present while the system was ready to respond, 67 responses were missed. The overall miss rate was therefore 10.6%, corresponding to a response rate of 89.4%.

Ultraviolet irradiance data were available for 24 benchmarked runs and ranged from 145 to 1441 µW cm^-2^. No significant association was detected between detection precision and mean ultraviolet irradiance over the sampled range (Spearman ρ = −0.06, p = 0.79).

Stimulus timing closely matched the programmed settings. The mean interval between confirmed detection and the activation command was 3.043 ± 0.014 s, compared with the programmed delay of 3 s. The mean stimulus duration was 4.001 ± 0.019 s, compared with the programmed duration of 4 s. No stimulation cycle differed from the programmed timing by more than 0.15 s.

The measured voltage across the shock grid remained within ±0.1 V of the selected voltage settings before and after the experimental runs. The feeder refill mechanism operated successfully throughout the experiment, with food solution delivered and maintained in the feeding cup.

## 4. Discussion

We have designed FloBuzz as a modular, field-deployable AFRFS to enable controlled bee–flower interaction experiments under natural conditions, coupling precisely timed, programmable aversive stimulation with reward delivery. The integration of a standardized feeder module, an electronically switchable shock grid, and a vision-triggered control pipeline (In-Trigger) allowed the system to operationalize closed-loop stimulus delivery. The electric shock stimulus is activated contingent on the bee’s presence at the feeder, rather than on a fixed schedule. Our design addressed a significant practical bottleneck in behavioral ecology by empowering behavioral assays on free-flying bees that were previously restricted to laboratory arenas.

Compared with the broader AFRFS literature, FloBuzz’s differentiator is that it is not primarily a reward-centric, cue-manipulative, or remotely operable AFRFS; instead, it adds a programmable nociceptive channel and implements contingent (visit-triggered) aversive stimulation under field conditions. Many AFRFS and artificial-flower approaches are optimized for standardized reward delivery, manipulation of multimodal floral cues, long-term repeated trials, and monitoring of visitation dynamics [2,4,11,13,34]. The advantage of FloBuzz is its explicit targeting of aversive paradigms in fields conventionally confined to laboratory settings [3,22,23,25,35,36]. Evidently, it complements field-associated aversive approaches based on predator cues or heat-based nociception [15–19]. FloBuzz complements these approaches by providing computer-vision-triggered electric stimulation within an automated feeding system that can be operated under field conditions.

The field experiment demonstrated that FloBuzz delivered controlled aversive stimulation during repeated outdoor trials and that bee occupancy of the feeder declined as the applied voltage increased. Mean occupancy decreased from 6.59 bees at 0 V to 3.61 bees at 12 V, corresponding to a 45.2% reduction. Consistent with this pattern, the block-adjusted dose-response model identified a significant negative relationship between voltage and mean bee occupancy. These results provided behavioral evidence that the stimulation delivered by FloBuzz produced a measurable deterrent response in visiting bees. Accordingly, the results should be interpreted as the validation of the system’s ability to deliver automated, voltage-dependent aversive stimulation rather than a demonstration of learning or other higher-order cognitive processes.

Treatment order and the absence of individual identification limit the interpretation of the field validation. The ascending blocks were conducted first, followed by the non-monotonic and descending blocks; thus, treatment sequences were grouped across the experimental period, and voltage treatments were not fully balanced across run positions. Although the negative voltage association persisted after adjustment for block and run position, this analysis cannot exclude carryover from earlier treatments or cumulative exposure across blocks. Furthermore, bees from five colonies shared one feeder, and individuals were unmarked, preventing identification of colony origin, repeated visits, and individual stimulation histories. The colonies therefore did not provide independent colony-level replication. Reduced occupancy may reflect immediate withdrawal, altered return behavior, or learned avoidance, which the present experiment cannot distinguish. Future studies should balance treatment sequences across days and combine individual identification with synchronized visit and stimulation records to quantify return probabilities and responses to repeated exposure.

The benchmark evaluation showed an overall detection precision of 91.9%, a miss rate of 10.6%, and stimulation timing closely matching the programmed delay and duration. No association between detection precision and mean ultraviolet irradiance was detected over the sampled range, but this result does not establish that detection performance is unaffected by lighting conditions. Our field observations indicated that these false positives were generally associated with moving shadows passing across the feeder and shock zone, particularly beneath vegetation where branches and leaves generated rapidly changing light patterns. Therefore, although FloBuzz can operate under variable outdoor light conditions, experiments should preferably be conducted in open areas with relatively uniform illumination and away from trees or other objects that generate moving shadows. This simple site-selection criterion may further improve detection precision and reduce unnecessary stimulus activation during field experiments.

A key advantage of FloBuzz is its combination of modular hardware that can be adapted to diverse feeder geometries and closed-loop triggering driven by real-time computer vision rather than manual operation. Furthermore, the In-Trigger module is species-agnostic and can be integrated into experimental setups for a wide variety of animals.

The present study establishes FloBuzz as a field-compatible platform for automated aversive stimulation in freely foraging bees. Its modular design and programmable control architecture provide flexibility for adapting the system to different experimental questions and behavioral paradigms. Future studies can build on this framework by incorporating additional stimuli, experimental conditions, and response measures to investigate more complex aspects of pollinator behavior under controlled yet ecologically relevant conditions.

## Supporting information

S1 Fig Custom PCB design of syringe pump control unit. (PDF)

S2 Fig Circuit diagram of the shock switch module. (PDF)

S3 Fig Flowchart of the In-Trigger module. (PDF)

S4 Fig Mean bee occupancy across successive voltage treatments within individual experimental blocks. Panels show the nine blocks in chronological order from left to right and top to bottom. Headings indicate the date, block number within that day, and treatment sequence. Block numbering restarts each day. Each point represents mean occupancy calculated from 60 counts recorded at 5-s intervals during a 300-s run. Colors and labels identify voltage treatments, and lines connect successive runs within each block. All panels use the same vertical scale.
(TIFF)

S1 Movie A movie showing 12V electric shock experiments. The moment when the electric shock is activated shown with a red circle on the video.
(MP4)

S1 Appendix Supplementary hardware and firmware description. (DOCX)
