# Supplementary material for: FloBuzz: A modular feeder system for automated aversive stimulation in free-flying bees
Source: PLoS One. 2026 Oct 26;21(10):e0361207. doi: 10.1371/journal.pone.0361207 (PMC13704823; doi:10.1371/journal.pone.0361207)
Supplement: Supplementary file 1 [file pone.0361207.s001.pdf]

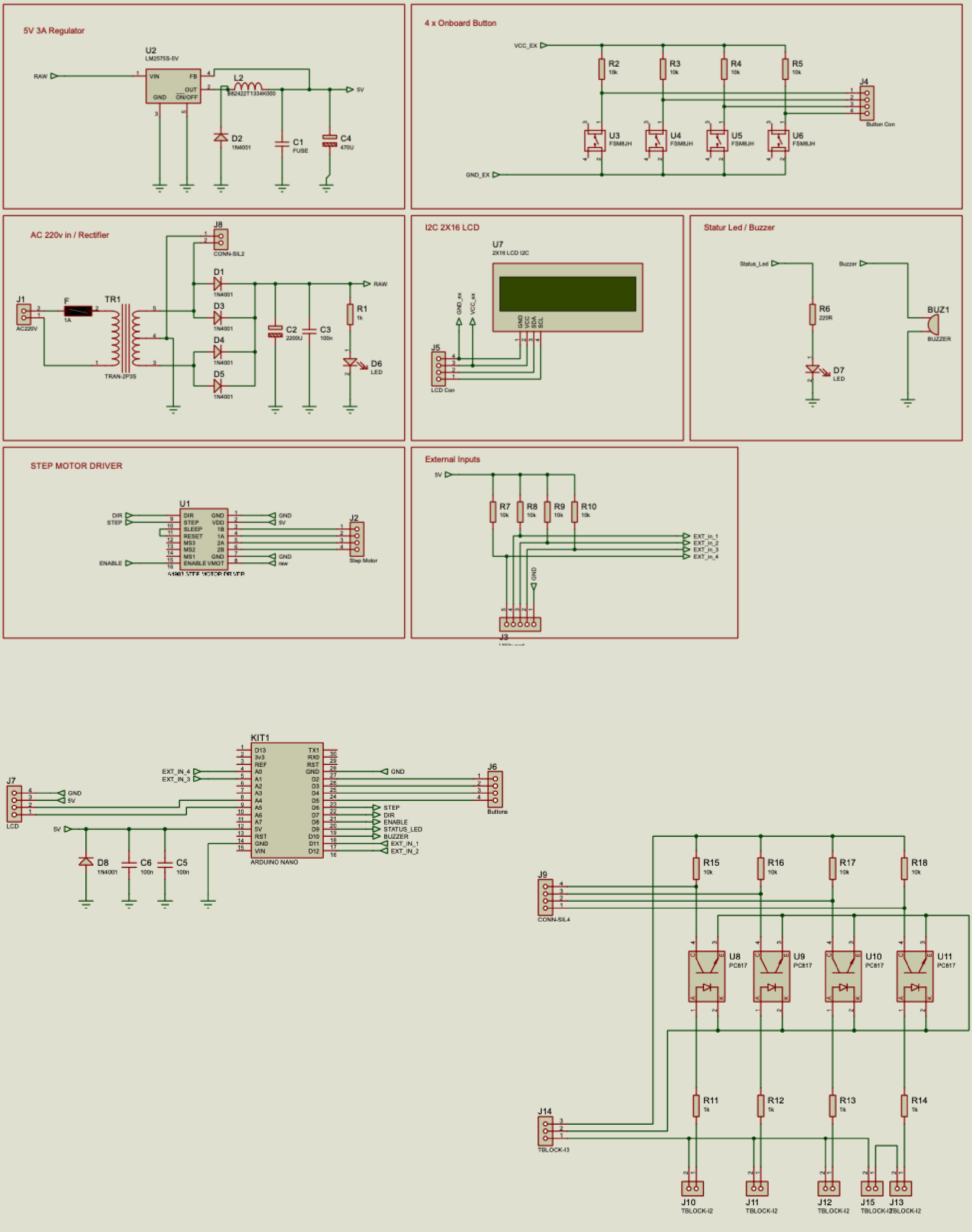

5V 3A Regulator
U2
LM2575S-5V
RAW
VIN
FB
OUT
GND
ON/OFF
L2
B82422T1334K000
5V
D2
1N4001
C1
FUSE
C4
470U
4 x Onboard Button
VCC_EX
GND_EX
R2
R3
R4
R5
10k
J4
Button Con
U3
U4
U5
U6
FSM8JH
AC 220v in / Rectifier
J8
CONN-SIL2
J1
AC220V
F
1A
TR1
TRAN-2P3S
D1
D3
D4
D5
1N4001
RAW
C2
2200U
C3
100n
R1
1k
D6
LED
I2C 2X16 LCD
U7
2X16 LCD I2C
GND
VCC
SDA
SCL
GND_ex
VCC_ex
J5
LCD Con
Statur Led / Buzzer
Status_Led
Buzzer
R6
220R
D7
LED
BUZ1
BUZZER
STEP MOTOR DRIVER
U1
DIR
STEP
SLEEP
RESET
MS3
MS2
MS1
ENABLE
VDD
1B
1A
2A
2B
VMOT
GND
5V
raw
J2
Step Motor
External Inputs
5V
R7
R8
R9
R10
10k
EXT_in_1
EXT_in_2
EXT_in_3
EXT_in_4
J3
KIT1
ARDUINO NANO
D13
3v3
REF
A0
A1
A2
A3
A4
A5
A6
A7
5V
RST
GND
VIN
TX1
RX0
RST
GND
D2
D3
D4
D5
D6
D7
D8
D9
D10
D11
D12
EXT_IN_4
EXT_IN_3
J7
LCD
GND
5V
D8
1N4001
C6
100n
C5
100n
J6
Buttons
STEP
DIR
ENABLE
STATUS_LED
BUZZER
EXT_IN_1
EXT_IN_2
J9
CONN-SIL4
R15
R16
R17
R18
10k
U8
U9
U10
U11
PC817
R11
R12
R13
R14
1k
J14
TBLOCK-I3
J10
J11
J12
J15
J13
TBLOCK-I2
