# Supplementary material for: FloBuzz: A modular feeder system for automated aversive stimulation in free-flying bees
Source: PLoS One. 2026 Oct 26;21(10):e0361207. doi: 10.1371/journal.pone.0361207 (PMC13704823; doi:10.1371/journal.pone.0361207)
Supplement: Supplementary file 2 [file pone.0361207.s002.pdf]

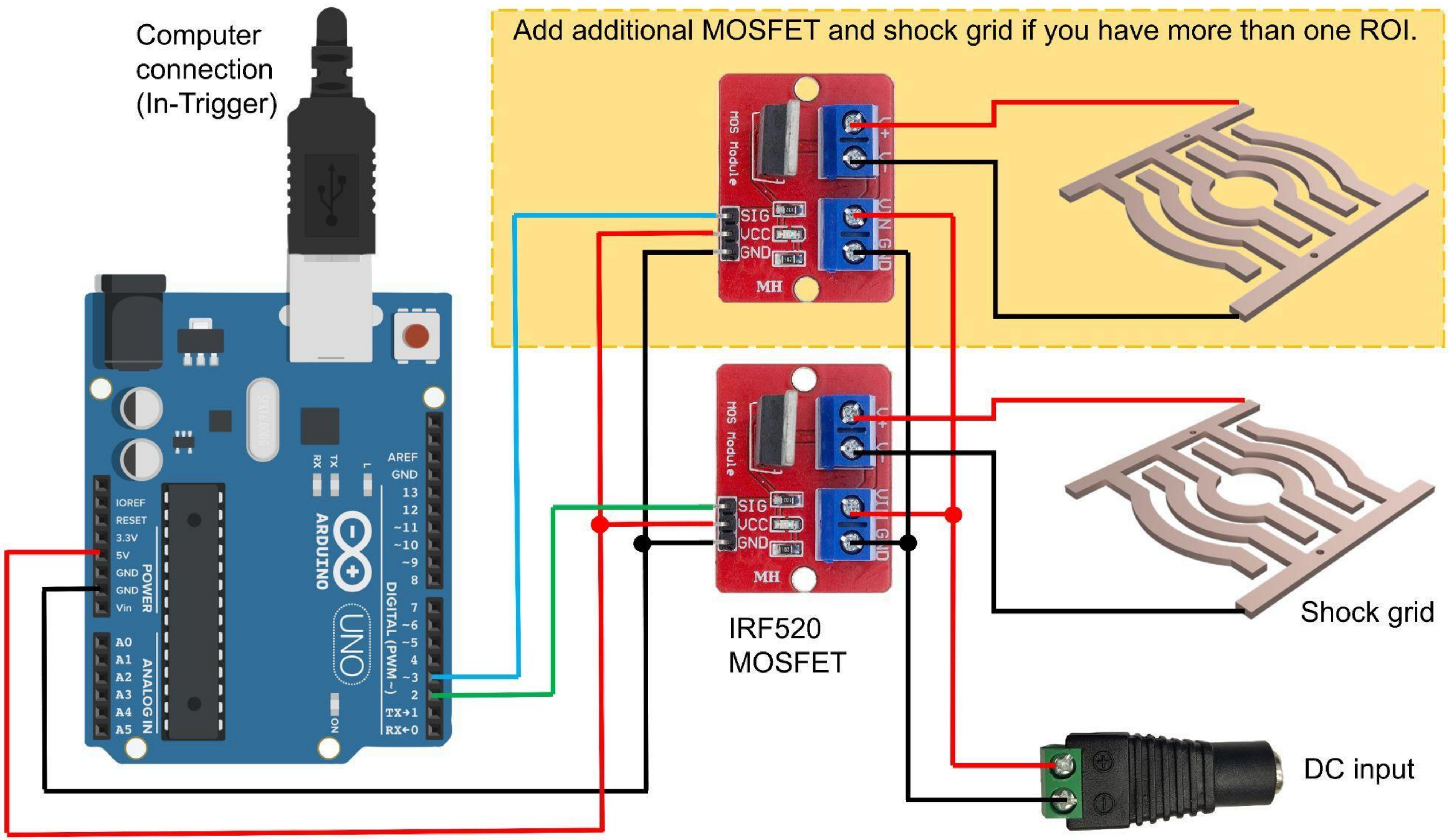

Computer connection (In-Trigger)
Add additional MOSFET and shock grid if you have more than one ROI.
SIG
VCC
GND
MH
IRF520 MOSFET
Shock grid
DC input
ARDUINO
UNO
IOREF
RESET
3.3V
5V
GND
GND
Vin
POWER
A0
A1
A2
A3
A4
A5
ANALOG IN
AREF
GND
13
12
~11
~10
~9
8
7
~6
~5
4
~3
2
TX→1
RX←0
DIGITAL (PWM~)
