# Supplementary material for: FloBuzz: A modular feeder system for automated aversive stimulation in free-flying bees
Source: PLoS One. 2026 Oct 26;21(10):e0361207. doi: 10.1371/journal.pone.0361207 (PMC13704823; doi:10.1371/journal.pone.0361207)
Supplement: Supplementary file 3 [file pone.0361207.s003.pdf]

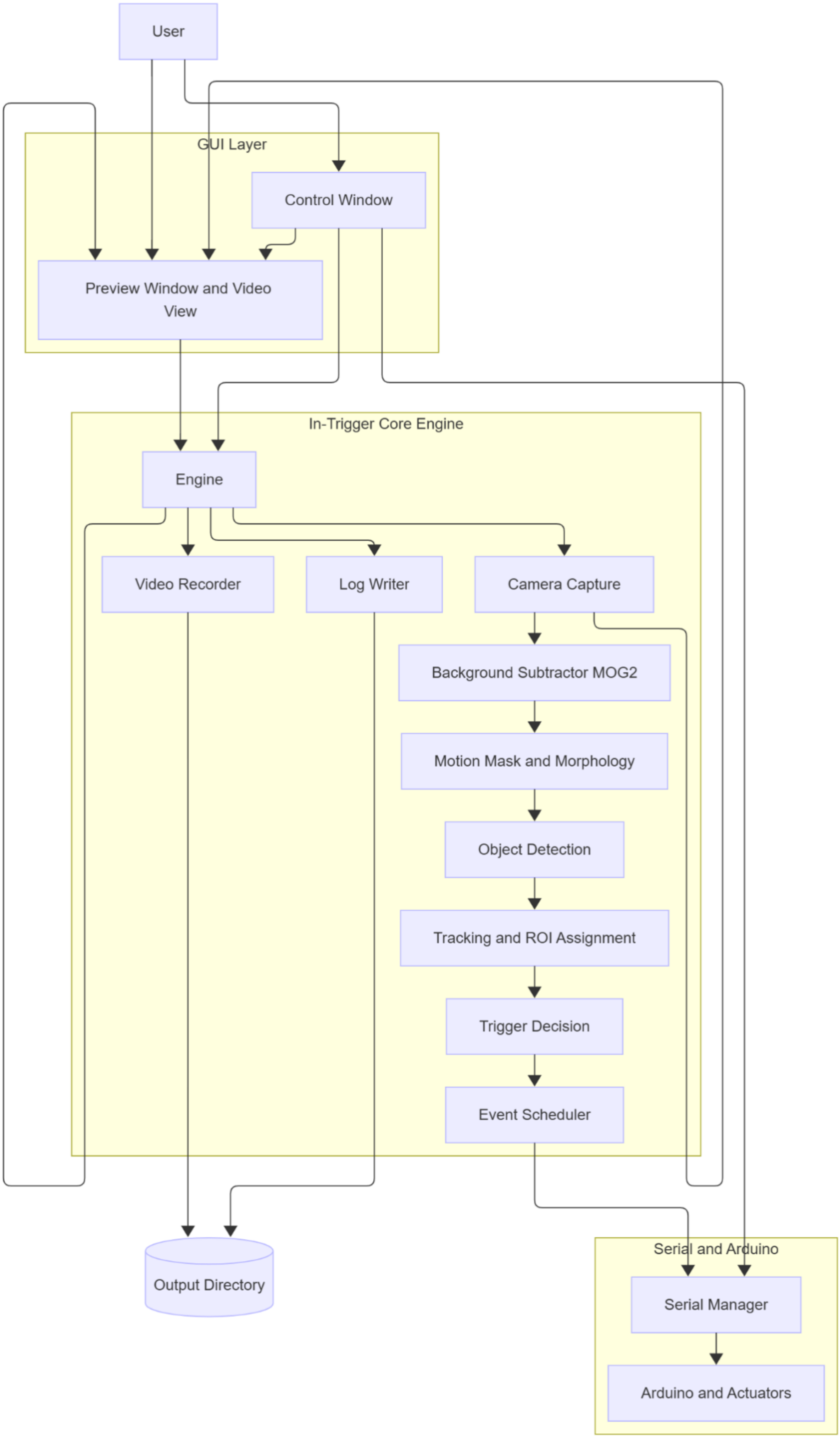

User
GUI Layer
Control Window
Preview Window and Video View
In-Trigger Core Engine
Engine
Video Recorder
Log Writer
Camera Capture
Background Subtractor MOG2
Motion Mask and Morphology
Object Detection
Tracking and ROI Assignment
Trigger Decision
Event Scheduler
Output Directory
Serial and Arduino
Serial Manager
Arduino and Actuators
